# The Effects of Ionizing Radiation on Gut Microbiota, a Systematic Review

**DOI:** 10.3390/nu13093025

**Published:** 2021-08-29

**Authors:** Ana Fernandes, Ana Oliveira, Raquel Soares, Pedro Barata

**Affiliations:** 1 Department of Nuclear Medicine, Centro Hospitalar Universitário de São João, E.P.E., 4200-319 Porto, Portugal; ana.ccoliveira@chsj.min-saude.pt; 2 Department of Biomedicine, Faculdade de Medicina da Universidade do Porto, 4200-319 Porto, Portugal; raqsoa@med.up.pt; 3 i3S, Instituto de Investigação e Inovação em Saúde, Universidade do Porto, 4200-135 Porto, Portugal; pbarata@ufp.edu.pt; 4 Department of Pharmaceutical Science, Faculdade de Ciências da Saúde da Universidade Fernando Pessoa, 4249-004 Porto, Portugal; 5 Department of Pathology, Centro Hospitalar Universitário do Porto, 4099-001 Porto, Portugal

**Keywords:** microbiome, microbiota, intestinal microbiome, gut microbiota, microflora, ionizing radiation, radiotherapy, radiation effects

## Abstract

Background: The human gut microbiota is defined as the microorganisms that collectively inhabit the intestinal tract. Its composition is relatively stable; however, an imbalance can be precipitated by various factors and is known to be associated with various diseases. Humans are daily exposed to ionizing radiation from ambient and medical procedures, and gastrointestinal side effects are not rare. Methods: A systematic search of PubMed, EMBASE, and Cochrane Library databases was conducted. Primary outcomes were changes in composition, richness, and diversity of the gut microbiota after ionizing radiation exposure. Standard methodological procedures expected by Cochrane were used. Results: A total of 2929 nonduplicated records were identified, and based on the inclusion criteria, 11 studies were considered. Studies were heterogeneous, with differences in population and outcomes. Overall, we found evidence for an association between ionizing radiation exposure and dysbiosis: reduction in microbiota diversity and richness, increase in pathogenic bacteria abundance (Proteobacteria and Fusobacteria), and decrease in beneficial bacteria (*Faecalibacterium* and *Bifidobacterium)*. Conclusions: This review highlights the importance of considering the influence of ionizing radiation exposure on gut microbiota, especially when considering the side effects of abdominal and pelvic radiotherapy. Better knowledge of these effects, with larger population studies, is needed.

## 1. Introduction

The human gut microbiota is defined as the microorganisms (bacteria, viruses, archaea, and protists) that collectively inhabit the lumen and the mucosal surface of the intestinal tract. The collection of all genomes of those microorganisms constitutes the intestinal microbiome [1,2].

Each individual gut microbiota composition is established early in life and is relatively stable over time. However, an imbalance of its composition (dysbiosis) can be precipitated by various exogenous and endogenous factors, such as significant changes in diet, infections, the use of antibiotics, or abdominal surgery [2,3]. Dysbiosis has been associated with a wide variety of pathologies, including gastrointestinal and nongastrointestinal diseases [1,2,4].

Ionizing radiation consists of energy capable of detaching electrons from atoms or molecules, thus ionizing them. It results from the decay of radionuclides (unstable atoms) and may take the form of electromagnetic waves (gamma (γ) or X-rays) or particles (alpha, beta, or neutrons) [5,6,7,8].

Interaction of radiation with matter results in indirect and direct effects, ranging from creating free radicals (mainly from water molecules) to altering the DNA molecule and its actual destruction [5,6,7,8].

As such, depending on its type, energy, and penetration, ionizing radiation may temporarily affect the function of molecules and atoms, lead to mutations that may be transmitted to the following generations, and, ultimately, lead to the destruction of cells [8,9].

Following the exposure, possible damage to the tissues depends on the radiation dose and tissue’s radiosensitivity. The most radiosensitive cells are those rapidly dividing, well-nourished, and with high metabolic activity [1,10,11]. Nevertheless, beyond certain thresholds, there are known expectable acute effects that will occur independently of the tissue [8].

It should be noted that some microorganisms are resistant to higher levels of ionizing radiation. Bacterial survival and adaptation to stressors include a complex network of regulation, including post-transcriptional regulators, such as small RNAs, that when adequately combined may enhance bacterial resistance to ionizing radiation. A better understanding of these mechanisms and which bacteria are more prone to be affected by ionizing radiation may prove helpful to predict and prevent dysbiosis [12,13].

Exposure may be natural or human-made. Daily, global natural exposure derives from naturally occurring radioactive materials and cosmic rays. Human-made sources of exposure result from nuclear power generation or, more frequently, from medical procedures, namely in radiology and nuclear medicine procedures and in radiotherapy treatments.

Despite its vast benefits, radiation from medical procedures can cause adverse effects [8,14,15], including frequent gastrointestinal toxicity during abdominal and pelvic radiotherapy [5,6,7]. Current evidence suggests that the gut microbiome influences radiotherapy efficacy [16,17] and radiation-induced gastrointestinal toxicity [5,7,18,19].

The relation between the gut microbiota and the pathogenesis of radiation-induced gastrointestinal toxicity is believed to be mediated through inflammatory processes, disruption of the epithelial barrier and intestinal permeability, epithelial repair and expression, and release of immune molecules in the intestine. Dysbiosis, whether caused by radiation or other factors, can influence both local and systemic immune responses. Research suggests that gut microbiota composition and diversity could be used as predictive biomarkers for radiotherapy outcomes, so further investigation is essential. Hence, we sought to systematically review the existing evidence of the effects of ionizing radiation on gut microbiota [20,21,22]. 

The aim was to conduct a systematic literature review of all studies involving human subjects that reported effects of ionizing radiation on gut microbiota, either performed in vivo or in vitro. Key outcomes were radiation-induced changes in the gut microbiota, namely in its composition, diversity, or richness/abundance.

## 2. Materials and Methods

### 2.1. Search Strategy and Selection Criteria

A systematic search was carried out using the following electronic databases: PubMed/MedLine (23/03/2021), EMBASE (16/08/2021), and Cochrane Library (17/08/2021). Additional articles were identified through the reference list from the included articles and relevant reviews. To ensure that studies had not been missed or wrongly excluded and the search was comprehensive, we searched gray literature, general search engines, and reference lists of included papers. 

**Figure 1:**
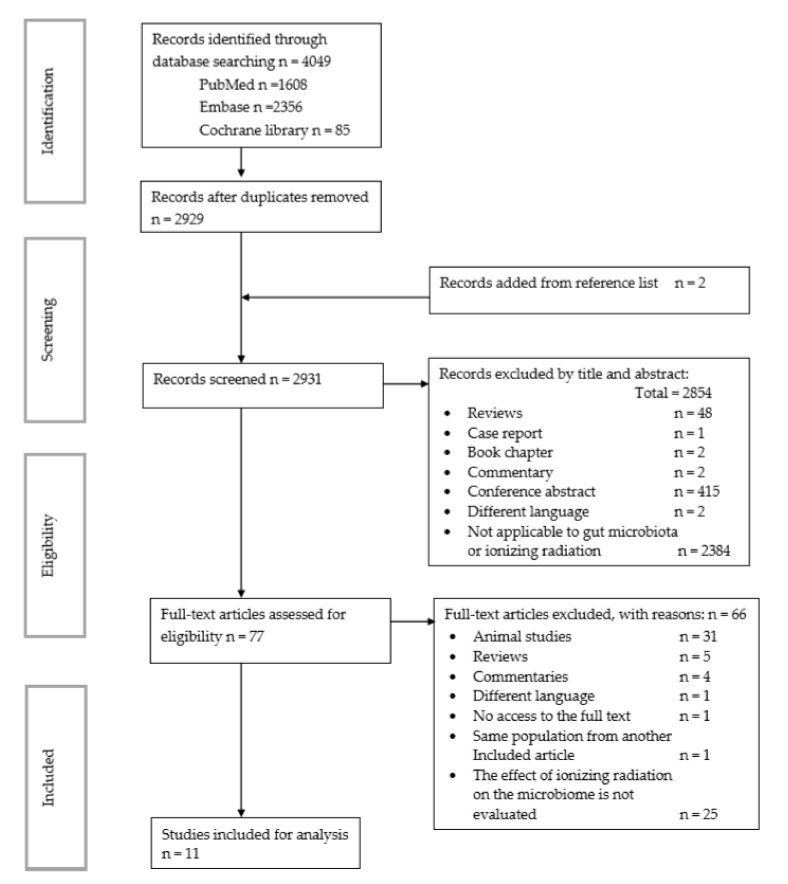
PRISMA flow chart search strategy.

This review was carried out following the Preferred Reporting Items for Systematic Reviews and Meta-Analyses (PRISMA) guidelines checklist (see Table S1). In addition, the review protocol was registered on the International PROSPERO review database on 5 November 2020: PROSPERO 2020: CRD42020210951 (https://www.crd.york.ac.uk/prospero/display_record.php?ID=CRD42020210951 (5 November 2020) (see Figure 1 for PRISMA diagram and Table 1, Table 2 and Table 3 for search terms). 

The PROSPERO database and Cochrane Library revealed no similar systematic reviews. All selected citations were exported from the databases to the reference management software EndNote X20 (Thompson Reuters, New York, New York, USA), and duplicates were excluded.

### 2.2. Inclusion and Exclusion Criteria

Inclusion criteria were defined using the following components: patient population (P): humans exposed to radiation; exposure of interest (I): ionizing radiation; comparator (C): before and after exposure of the same subject or with controls; outcome (O): the changes in the gut microbiome following exposure to radiation; study design (S) of interest: randomized controlled trials and prospective and retrospective observational cohort studies. Initially, the authors searched for studies in human and animal subjects, but after analyzing the significant differences between the studies, the authors decided to separate the review. Therefore, this review is focused on human studies.

### 2.3. Study Selection and Data Extraction

All relevant peer-reviewed journal articles in English, Portuguese, and Spanish indexed until March 2021 were identified. A combination of search terms was used: microbiome, gut microbiota, radiotherapy, ionizing radiation, 16S rRNA, and microflora (Table 1, Table 2 and Table 3). The final search was performed on 17 August 2021 by two authors (AF and PB).

According to the defined inclusion and exclusion criteria, relevant studies were independently screened by two reviewers (AF and PB) based on title and abstract. All decisions were recorded on a spreadsheet. 

All studies that did not fulfill the defined PICOS characteristics, conference papers, abstracts, and articles for which we could not obtain the full text were excluded.

Full-text papers of all available eligible studies were obtained, and the two reviewers independently screened and selected papers a second time. 

A tabular summary with the following variables that were extracted from each eligible study was developed for this review: first author name; date of publication; study design; number of patients and controls; radiation exposure characteristics; type, number, and time point of samples; and the most relevant findings (Table 4).

### 2.4. Risk of Bias in Individual Studies 

Two reviewers (AF and PB) assessed the risk of bias of each study independently, with disagreements resolved by consensus. The risk of bias was assessed as described in the Cochrane Handbook [28] by recording the methodology used. 

The quality of nonrandomized studies was assessed by using the Newcastle–Ottawa Scale. The quality of the studies was examined for (a) selection, (b) comparability, and (c) outcome (Table 5) [29].

The quality of the randomized controlled study was analyzed as recommended by the Cochrane Collaboration [28], using the following domains: sequence generation, allocation concealment, blinding, incomplete outcome data, selective outcome reporting, and other sources of bias (Table 6).

No formal statistical analysis was undertaken due to the small number of eligible studies and the heterogeneity of the data and outcomes presented.

## 3. Results

### 3.1. Search Results

The database search resulted in 4049 titles (August 2021): 1608 relevant abstracts were identified through PubMed, 2356 through EMBASE, and 85 through Cochrane Library (Figure 1). After removing duplicates and including two papers identified from the reference list, 2929 papers were screened for inclusion based on title and abstract. Of these, 2854 were excluded based on title and abstract (415 were conference abstracts, 2 were book chapters, 48 were reviews, 2 were commentaries, 2 were articles in different languages unreadable by authors, 1 was a case report, and 2384 were not focused on gut microbiota and/or ionizing radiation). Full texts of the remaining 77 studies were carefully assessed, and further 66 were excluded (31 concerned animal studies, 25 did not report the effect of ionizing radiation in microbiota [30,31,32], 5 were reviews [33], 4 were commentaries [34], 1 was in a different language unreadable by authors [35], and the authors could not access to the full text of 1 article). Thus, finally, a total of 11 studies were included in this review.

### 3.2. Study Characteristics

Eleven analytic studies were included, 1 randomized control trial and 10 prospective cohort studies. A summary of the study characteristics and key findings is presented in Table 4. The analyzed studies were quite heterogeneous regarding patient characteristics, study methodology, and evaluated outcomes. The 11 studies recruited 424 participants, of which 361 were exposed to ionizing radiation and 63 were controls. The median number of participants exposed to ionizing radiation was 22 (range 5–115). Healthy controls were enrolled in five studies, ranging from 2 to 31 participants [5,7,17,24,27]. The patients’ demographics of each study are summarized in Table 4. The age range of participants was 3–79 years. Two studies included 98 participants younger than 18 years old [24,27], including the study published by Sahly et al. that analyzed the gut microbiota of only three children with rhabdomyosarcoma and two controls [27]. One study did not provide complete information regarding the participants’ demographics besides their age range [24]. Of the remaining 10 studies, five only included women [5,18,23,25,26], and one included only male participants [27]. 

Most studies evaluated the effect of ionizing radiation from a medical exposure (pelvic radiotherapy) [5,7,16,17,18,19,23,26], except the study of Sajjadieh et al., who conducted a study to evaluate the gut microbiota changes in 75 rural patients aged between 3 and 18 who lived in a contaminated area at a distance of 60 to 90 km from the Chernobyl Nuclear Power Plant and were exposed to natural environmental radiation and presented abdominal/gastrointestinal symptoms. Additionally, an older method of microbiome analysis, relying on bacterial culture colony-forming units, was used [24]. 

García-Peris et al. and Cuzzolin et al. also used older microbiota detection methods for taxa identification: fluorescent in situ hybridization using genus-specific probes for Bifidobacterium and Lactobacillus (Bif164 and LAC158, respectively) and agar-based culture for enteric bacteria, respectively [23,25]. 

The remaining seven studies were found to be more homogeneous. These prospective cohort studies were conducted in 237 patients, 128 gynecological cancer patients, and 109 colorectal cancer patients treated with pelvic radiotherapy, with doses ranging from 44.0 to 50.4 Gy, mostly five times a week for five weeks and used 16s rRNA for taxa identification [5,7,16,17,18,19,26]. Nevertheless, one of the studies did not report the doses to which the patients were exposed [18]. 

The number of obtained samples per patient was very heterogeneous between studies: one sample [24], two samples [7,16,17,26], three samples [27], four samples [5,18,25], and five samples [19,23].

The time points of the sampling collections within the studies were also found to be quite heterogeneous. Ten studies collected samples before and after exposure. The first samples were collected a week before, the previous days, or immediately before treatment initiation. The collection of samples after exposure varied from immediately after exposure to three months after. 

In six studies, patients were concomitantly treated with chemotherapy [5,16,17,18,19,27], and in four of those studies, patients were also concomitantly treated with antibiotics [17,18,19,27]. Only five studies were conducted without using concomitant chemotherapy or antibiotic therapy [7,23,24,25,26]. Most studies considered treatments with immunosuppressor drugs, prebiotics, or probiotics but failed to mention other concomitant medications or comorbidities.

### 3.3. Sampling and Microbiota Analysis

Overall, the studies included in this review characterized the gut microbiota through fecal samples, except for the study of El Alam et al., which used rectal swabs [19]. Fecal samples are considered the most convenient and the most frequently used collection method in large-scale studies. They are noninvasive and have long been considered as an accurate representation of the distal gut microbiota. Fecal samples have the disadvantages that they might contain inactive bacteria, bacteria from other gastrointestinal tract compartments, and less controlled sampling variables, compared with biopsy [36]. Rectal swabs, despite being easier to sample, have the disadvantages of no visual aid to pinpoint areas of interest, limited biomass for host studies, more discomfort than fecal sampling, and potential contamination with skin bacteria [36]. The methods used to characterize the microbiota also varied throughout the studies. Most of the reviewed studies opted for 16S rRNA-based sequencing; however, the studies performed before 2013 used culture-based microbiota assessment techniques [23,24,25]. Even though there are several software packages available for microbiome data analysis, most of the reviewed studies chose to use QIIME [5,26,27] and/or MOTHUR [5,7,16] and a reference database for taxonomic classification, such as SILVA [7,16,18], Greengenes [26], Ribosomal Database Project [5,17], or UNITE [36]. Richness (number of OTUs/species) and diversity (alpha diversity (within a single sample) and/or beta diversity (between two samples)) were parameters assessed in most of the reviewed studies. There are various methods available for calculating alpha diversity, which considers the richness of the sample and/or the evenness (relative abundance of different OTUs/species and their even distribution). Commonly used methods included in our studies were the number of observed species, Chao1 index (estimates the richness), Shannon’s index, and Simpson’s index (richness and evenness). For beta diversity, Bray–Curtis, unweighted UniFrac, and weighted UniFrac were used [36,37,38]. 

### 3.4. Findings

The analyzed studies suggest that ionizing radiation causes significant changes in the composition, diversity, and richness of the gut microbiota. Key findings of the studies are organized in Table 7.

#### 3.4.1. Diversity and Richness Analysis

Seven studies demonstrated that ionizing radiation decreases richness, as measured by the number of OTUs, Chao1 index, and richness index [5,7,17,18,19,26,27]. Only one study reported that the richness and diversity remained unchanged [16]. The three studies that used cultured-based methods could not assess these parameters [23,24,25]. Other parameters such as α-diversity, as measured by Shannon index and Simpson index, also decreased after ionizing radiation exposure in most studies [5,7,18,19,26,27]. The only exception was observed by Yi Y et al.; despite decreases in other parameters (Chao1 index and richness index), they reported an increase in Simpson index, even without statistical significance (p=0.32) [17].

#### 3.4.2. Gut Microbial Composition

All studies reported changes in the microbiota composition after exposure to ionizing radiation, but the methodology of reporting of results was highly variable among them; some only analyzed alterations at phylum or genus level. In addition, only three studies analyzed species level [5,16,23]. The culture-based studies had limited results of the specific bacteria taxa analyzed. 

Regarding the composition of the gut microbiota, one of the most consistent findings was an increase in the relative abundance of the bacteria from the Proteobacteria phylum following radiation exposure. Sahly et al. and El Alam et al. reported an increase in the Proteobacteria [19,27], and there was a fluctuating pattern in the findings of Nam et al., initially increasing after the first session and then decreasing after the fifth and in follow-up samples [5]. Other taxonomic levels from the Proteobacteria phylum also increased after exposure: Gammaproteobacteria class [19], the order Pasteurellales [18,19], the family *Pasteurellaceae* and *Haemophilus* genera [19], and the genera *Serratia* [26].

One study showed an increase in the Actinobacteria phylum [27], and Nam et al. found a fluctuating pattern, increasing after the first radiotherapy session and decreasing after the fifth session and in the follow-up sample [5]. *Bifidocaterium*, the most important genus from the Actinobacteria phylum, decreased in three studies [24,25]. 

The abundance of the Fusobacteria phylum significantly increased in one study [5]. The relative abundance of the order Fusobacteriales increased in one study [18], and the family *Fusobacteriacea* was also significantly increased in one study; conversely, the genus *Fusobacterium* showed a significant decrease reported only in one study [5,17]. 

The relative abundance of unclassified bacteria showed significant differences after IR exposure in two studies, increasing following radiotherapy treatments [5,7].

Three studies showed a decrease in the Firmicutes/Bacteroidetes ratio (F/B ratio) (decrease in the Firmicutes phylum and increase in the Bacteroidetes phylum) [5,7,27]. 

Results observed for the phylum Bacteroidetes were mixed. The relative abundance decreased in one study [19]. Conversely, Nam et al. reported decreases during radiation therapy but large increases in the follow-up samples, while two other studies reported relative abundance increases [5,7,27]. The genus *Bacteroides*, from the Bacteroidetes phylum, also showed mixed outcomes, increasing in two studies [7,27] and decreasing in two other studies [17,26]. 

Nam et al. reported a decrease of 10.1% in the Firmicutes phylum [5]. Regarding the taxa from the Firmicutes phylum, the order Lactobacillales increased in one study [18]. The genus *Lactobacillus* decreased in two studies [24,25] and increased in one study [17]. The *Lactobacillus murinus* species decreased in one study [5] and the *Lactobacilli aerobi* and *anaerobi* species both decreased in one study [23]. *Oscillibacter* significantly decreased in two studies [7,17]. The relative abundance of the *Faecalibacterium* genus decreased in three studies [7,17,27], and *Faecalibacterium prausnitzii* was reported to decrease in another study [16].

## 4. Discussion

This review provides a detailed overview of the clinical studies describing the effect of ionizing radiation on gut microbiota composition.

Nevertheless, there were several limitations of the study. Most studies had a reduced number of participants, and in some of the larger trials, not all participants provided all the samples. For example, El Alam et al. recruited 58 participants, but only 5 provided samples at every time point [19].

Remarkably, the dosage and duration of radiation exposure might have a significant impact on the results. Sheikh et al. [24] analyzed the influence of ambient exposure, and the remaining studies analyzed the influence of pelvic radiotherapy treatment. Most studies used comparable doses and sessions. 

Three studies used culture-based methods that limited the information to two and four genera [23,24,25]. The remaining studies used 16S rRNA sequencing to characterize the taxonomic distribution and diversity of gut microbiota. 16S rRNA is a cost-effective semiquantitative method [2]. 

Despite being the most commonly utilized method, 16S rRNA presents some disadvantages. For instance, the accuracy of identification depends on the extent of the reference database, the primers used for 16S rRNA amplification may lead to potential biases, and the resolution power is only at the species level, but most studies only analyzed genus level [39].

The studies that used 16S rRNA clustered reads into operational taxonomic units (OTUs), which are grouped based on 97% DNA sequence similarity [36]. The OTU clustering allows diversity analyses, taxonomic classification through databases, and a variety of statistical analyses to assess the differences in distribution and abundance between samples and groups [36].

Functional gut microbiota can be assessed using metagenomics, metatranscriptomics, metaproteomics, and metabolomics. Shotgun metagenomics is a quantitative method that provides a vast amount of functional information, identifying the strain level (low-level taxonomic rank describing genetic variants or species subtypes). However, it is very costly [2]. None of the reviewed studies in this review utilized metagenomic or metatranscriptomic shotgun sequencing. 

The studies with a higher number of participants had major potential biases. Sajjadieh et al., who recruited a total of 95 participants, used culture-based techniques and only quantified the environmental radiation exposure at that moment [24]. García-Peris et al., who recruited 31 patients, did not include controls as a comparison group and used culture-based techniques to evaluate only two genera [25]. El Alam et al., who recruited 58 participants, had only five participants providing samples at all time points [19]. Finally, 115 participants were recruited by Yi Y et al.; however, samples were only collected three days after the exposure, neglecting the long-term effects. Mitra et al. (n = 35), Wang A et al. (n = 15), Cuzzolin et al. (n = 15), Wang Z et al. (n = 18), and Shi et al. (n = 22) also did not evaluate long-term effects. These are further limitations of these studies, given that the studies that had long-term evaluations reported gradual changes and significant differences in the follow-up sample [5,19,27].

Differences in diversity, richness, and taxonomic composition varied across studies, with multiple different outcome measures. Nevertheless, some concordant results emerged. Overall, gut microbiota diversity and relative abundance of individual bacterial taxa were affected after exposure to ionizing radiation. Most studies showed a decrease in diversity (especially alpha diversity), implying the development of a dysbiotic gut microbiota associated with several diseases as observed in the literature [40].

Regarding composition, the analyzed studies confirmed that the human gut microbiota is mainly composed of two bacterial phyla, Firmicutes and Bacteroidetes (usually more than 90%), and other less abundant phyla including Proteobacteria, Actinobacteria, and Verrucomicrobia [4,41,42].

Although differences in taxonomic composition varied across studies, one of the most consistent findings was the increased relative abundance of Proteobacteria following exposure to ionizing radiation. Proteobacteria phylum is composed of Gram-negative bacteria, including the well-known pathogenic genera *Escherichia*, *Salmonella*, *Helicobacter,* and *Legionellales*, and has been associated with inflammation, being a sign of dysbiosis [43,44].

Another consistent finding was the decreased relative abundance of the *Faecalibacterium* genus after exposure. The species *Faecalibacterium prausnitzii* (previously known as *Fusobacterium prausnitzii*) is one of the most abundant bacteria of the healthy human gut microbiota and is one of the most essential bacteria that produce butyrate and other short-chain fatty acids [45]. Its depletion has been arguably associated with inflammatory bowel disease [46].

Another noteworthy finding was an increased relative abundance of the bacteria from the Fusobacteria phylum, which are known to be associated with an extensive spectrum of infections [47].

Three studies reported a decrease in the Firmicutes-to-Bacteroidetes ratio [5,7,27]. The relationship between these two dominant phyla has been associated with several pathological conditions, including obesity [48]. However, the F/B ratio only takes into account a high-level taxonomic rank. It, therefore, is considered not reliable by more recent studies that evaluated other taxonomic levels (genus, species, or strain), suggesting that the complexity of how the gut microbiome modulates those diseases is far more complex than an imbalance of these two phyla [49].

*Bifidobacterium* and *Lactobacillus* genera are well known to exhibit probiotic effects and have shown to be beneficial for the host, being used in clinical practice for gastrointestinal diseases [24,25,50]. Two of the studies performed in culture growth-based methods reported decreases in abundance of the genera *Bifidobacterium* and *Lactobacillus* [24,25], and Cuzzolin et al., who also used a culture growth-based method, reported a decrease in *Lactobacilli aerobi* and *anaerobi* species [23]. Conversely, Yi Y et al. reported an increase in *Lactobacillus* [17].

*Bacteroides* are the most predominant anaerobes in most humans and tend to be the most abundant bacterial genus. They are known to have an essential role in the hydrolysis and fermentation of exogenous fiber and endogenous mucins, in the deconjugation of bile acids, and in the production of acetic and lactic acids [51,52]. Additionally, they have a role in stimulating the immune system, inducing the production of IL-2 by macrophages and B cells [24,53]. Generally, they tend to have a beneficial role in the gut, but when they go to another location, they can cause significant infections [53]. The analyzed studies reported mixed results: increases in relative abundance in two studies [7,27]; decreases in two other studies [17,26].

### 4.1. Limitations of the Studies

Overall, few high-quality studies were available, and several limitations were identified as the quality, methodology, and reporting of outcomes were highly variable among included studies. Therefore, the combined analysis of the selected studies presented conflicting results across a multitude of outcome measures. 

The primary limitation is that most trials were small in sample size (mean 22; range 5–115) and single-center trials, which may condition the study results and interpretation, as a small number of patients may fail to account for interindividual differences within the study population. Besides, information regarding the number of patients treated, eligibility, selection criteria, and recruitment timescale was rarely provided. Finally, only five studies used healthy volunteers as controls, and most studies did not describe their selection methods, include demographic information, account for possible confounders, or consider possible gut microbiome variability throughout time.

The inclusion of populations with different characteristics is also noteworthy: children vs. adults, inclusion of only female or male participants, and different types of tumors. It is well established that the diversity and composition of the gut microbiota are age-related [2], and sex differences in the gut microbiota composition have been recognized [54]. The possible role of sex and age on the microbiota and its impact is not acknowledged in any included studies. 

Another relevant limitation is that information regarding comorbidities, previous exposure to other sources of radiation, and concomitant medications (besides antibiotics, immunosuppressors, and prebiotics) is seldom mentioned in most studies, which makes it more complex to separate cohort effects from within-subject characteristics and could contribute to the heterogeneity of findings across the studies.

Most studies included patients with concomitant chemotherapy and/or antibiotic therapy, which are known to affect the human gut microbiota [55,56]; thus, it is not possible to isolate the unique effect of radiation.

All studies used fecal samples or rectal swabs, which may not fully represent the structure of the whole gut microbiota. Regarding detection methods, another limitation is that three studies used culture-based methods, and the remaining used 16S rRNA, so most studies only reported at the genus/phyla level; only three studies reported at the species level.

Finally, the number and time points of collection of the fecal samples also varied among the different studies, and these factors are known to affect the results regarding the gut microbiota composition.

### 4.2. Limitations of the Review

Due to the small number of studies found and the heterogeneity of the included study subjects, ionizing radiation exposure, and reporting methods, a meta-analysis was not performed.

One of the most significant limitations of this review was the heterogeneity among studies, especially regarding sample size, population characteristics, and sample time points.

## 5. Conclusions

This review highlights the importance of considering the effects of ionizing radiation exposure on the human gut microbiota, especially when abdominal and pelvic radiotherapy is being planned. The studies included herein demonstrated that dysbiosis develops after ionizing radiation exposure. It is important to note that there was high variability in the study population and in sampling time points in all included studies, which renders comparisons of the multiple findings rather tricky.

The most consistent and convincing evidence was that, after ionizing radiation exposure, diversity and richness are reduced, whereas pathogenic bacteria abundance, such as Proteobacteria and Fusobacteria, is increased. In addition, the abundance of the *Faecalibacterium* and *Bifidobacterium*, known to be beneficial bacteria, is decreased. These findings should be more explored and taken into account, especially when considering the side effects of medical treatments and further embracing prophylactic/therapeutic attitudes.

### Future Directions

Given the small sample sizes, the results are exploratory and should be interpreted cautiously. Only 11 studies were included in this review; the evidence regarding the effects of ionizing radiation on human gut microbiota calls for further studies, and the interpretation of the results should consider the several limitations listed above.

High-quality, large-scale trials should be carefully designed to determine the role of ionizing radiation in dysbiosis. More extensive studies, better-designed studies, and longer follow-up periods are needed to understand the process better.

Current evidence suggests that the gut microbiota is directly related to ionizing radiation-induced gastrointestinal toxicity and radiotherapy efficacy. A better understanding of the systemic effects of ionizing radiation and their relation to the gut microbiota is essential. Baseline gut microbial characteristics may serve as predictive tools to identify patients more likely to benefit from cancer treatments.

Future prospective longitudinal studies with larger samples will allow more complex models that account for important factors such as demographics, chronic medications, exercise, diet, and biological factors that might impact the gut microbiota composition.

## Figures and Tables

**Table 1:** Literature search algorithm—PubMed.

Search Number	Search Terms
Search #1	“microbiota” OR “gastrointestinal microbiome” OR “microbiome” OR “16s rRNA”
Search #2	“radiation” OR “radiotherapy”
Search #3	Search #1 AND Search #2

**Table 2:** Literature search algorithm—EMBASE (via OVID).

Search Number	Search Terms
Search #1	“microbiota” OR “gastrointestinal microbiome” OR “microbiome” OR “16s rRNA” OR “microflora”
Search #2	“radiation” OR “radiotherapy”
Search #3	English OR Spanish OR Portuguese
Search #4	Search #1 AND Search #2 AND Search #3

**Table 3:** Literature search algorithm—Cochrane Library.

Search Number	Search Terms
Search #1	“microbiota” OR “gastrointestinal microbiome” OR “microbiome” OR “16s rRNA” OR “microflora”
Search #2	“radiation” OR “radiotherapy”
Search #3	Search #1 AND Search #2

**Table 4:** Summary of study characteristics, demographics, radiation type, sample collection and analysis, and main findings of the eligible studies included.

Author, Year / DOI	Study Design	Participant Demographics: N; Sex (M:F); Age; Type of Cancer/Other	Type of Radiation	Microbiome Assessment Method / Software for Sequencing and For Identification	Type of Sample / Number of Samples	Main Findings	Antibiotic Used as Exclusion? / Comments
**Cuzzolin et al., 1992** [23] **/** **10.1080/1120009x.1992.11739160**	Prospective cohort	N = 15 (0:15) / 45–79 years / Gynecological cancer	Pelvic RT / 4000 cGy in 4 to 5 weeks overall 175–200 cGy daily 5 days per week	Culture counts / Agar-based methods	Fecal / 5 samples: - 1 before - 4 after irradiation fractions	- *Escherichia coli*—significant decrease after the 3 first RTs, *p* < 0.05 - *Aeromonas hydrophila*—significant decrease after the 2 first RTs, *p* < 0.05 - *Peptococcus* and *Peptostreptococcus* spp. and *Fusobacterium nucleatum*—significant decrease following the first radiation exposure, *p* < 0.05 - *Enterococcus faecium 1; Lactobacilli aerobi* spp.*,* total anaerobes, *Lactobacilli anaerobi spp.*—significant decrease after total dose RT, *p* < 0.05 - *Clostridium* spp. *(Cl. histolyticum, Cl. bifermentans, Cl. sporogenes)—*growth was observed in some patients - Fecal enterotoxin - not influenced by multiple radiations	Yes / Other exclusion criteria: cytotoxic chemotherapy
**Sajjadieh et al., 2012** [24] **/** **PMID: 23400266; PMCID: PMC3564093**	Prospective cohort	N = 75 Control group n = 20 / 4–18 years / Living in a contaminated area near Chernobyl	Ambient radiation / Internal whole-body radioactivity Cs-137 measured by γ-ray spectrometry	Bacterial culture; colony-forming units / CPLX agar Bifidobacterium; LBS agar Lactobacillus; COBA agar Enterococcus; DHL agar Enterobacter	Fecal / 1 sample	- Dysbiosis in 81.3% - *Enterobacter*—inconsistency found in the article between text and tabulated results: significantly decreased compared to control/increased in table, *p* < 0.01 - *Enterococcus*—significantly decreased compared to control, *p* < 0.01 - *Lactobacillus*—significantly decreased compared to control, *p* < 0.01 - *Bifidobacterium*—significantly decreased compared to control, *p* < 0.01	Yes / -
**García-Peris et al., 2012** [25] **/** **10.3305/nh.2012.27.6.5992**	RCT	N = 31 (0:31) / 36–77 years (median 59) / Gynecological cancer	Pelvic RT / 52.2 Gy 1.8 Gy/day 5 times a week 29 sessions	Culture counts / Fluorescent in situ hybridization, genus-specific probes (Bifidobacterium: Bif164 and Lactobacillus: LAC158)	Fecal / 4 samples: - 7 days before RT - 15 days after RT - At the end of the treatment - 3 weeks after RT	- *Lactobacillus*—significant decrease, *p* < 0.05 - *Bifidobacterium*—significant decrease, *p* < 0.05	Yes / Other exclusion criteria: previous RT; previous or adjuvant QT; immunosuppressive
**Nam et al., 2013** [5] **/** **10.1371/journal.pone.0082659**	Prospective cohort	N = 9 (0:9) Control N = 6 / 35–63 years / Gynecologic cancer (cervix and endometrium)	Pelvic RT / 50.4 Gy 5 times a week 5 week period 25 fractions	16S rRNA V1/V2 / QIIME MOTHUR UPARSE / Ribosomal and SILVA databases	Fecal / 4 samples:- 1 week before - After the first RT - At the end of the fifth RT - 1–3 months after final RT	- Shannon index—decrease, *p* = 0.220 - Number of unique sequences—decrease 10.4%, *p* = 0.06 - Estimated OTUs significantly decreased through the radiotherapy, *p* = 0.04 - Actinobacteria and Proteobacteria fluctuating pattern: increased after 1 RT session, decreased after the fifth and in the follow-up samples - Firmicutes steadily decreased 10% through radiotherapy, *p* = 0.09 - Fusobacteria increased 6.0 times, *p* = 0.05 - Bacteroidetes relative abundance gradually decreased during RT, but largely increased at T3 - Unclassified bacteria increased 9.9%, *p* = 0.04 - * Eubacteriaceae* —significantly decreased, *p* < 0.032 - * Fusobacteriaceae* —significantly increased at T2 - * Streptococcaceae* —significantly increased at T1, *p* < 0.05 - * Veillonellaceae* and *Enterococcaceae*, *Lactobacillales bacterium,* and *Butyrate-producing bacterium*—unchanged at T0, T1, and T2. Significant decrease from T0 to T3, *p* = 0.050 - *Ruminococcus*—slightly increased at T1 and eliminated at T2. Three ruminoccocal microorganisms were identified again at T3. In addition, some species-level taxa included in the same genus showed opposite patterns of variation (increment/decrement). - * Ruminococcus* sp. *CO28, Roseburia* sp. *DJF VR77, Ruminococcus* sp. *CO41,* and *Lachnospira pectinoschiza*—increase T0 vs. T1, *p* = 0.001 - * Weissella confuse, Enterobacter* sp. *mcp11b, Klebsiella pneumonia,* and *Adlercreutzia equolifaciens*—decrease 0.1% T0 vs. T1, *p* = 0.001 - * Butyrate-producing bacterium* SS2/1—increase 2.9% T0 vs. T2, *p* = 0.009 - * Ruminococcus callidus* —decrease 1.0% T0 vs. T2, *p* = 0.03 - * Dialister* sp. *E2 20* —decrease 1.0% T0 vs. T2, *p* = 0.013 - * Human intestinal firmicute CB47* —increase 3.5% T0 vs. T2, *p* = 0.025 - * Eubacterium eligens* —decrease 0.7% T0 vs. T2, *p* = 0.032 - * Eubacterium hallii* —decrease 0.1% T0 vs. T2, *p* = 0.041 - * Actinomyces odontolyticus* —decrease 0.1% T0 vs. T2, *p* = 0.046 - * Lactobacillus murinus* —decrease 0.1% T0 vs. T2, *p* = 0.039 - * Clostridiales bacterium DJF CP67* —increase 0.2% T0 vs. T2, *p* = 0.009 - *Clostridium sp. BGC36* was eliminated—decrease 0.1% T0 vs. T3, *p* = 0.001 - * Prevotella stercorea* —decrease 0.3% T0 vs. T3, *p* = 0.001 - *Ruminococcus* sp. DJF VR52, *Prevotella copri*, Ruminococcus sp. CO28, *Butyrate-producing bacterium* T1-815, *Roseburia inulinivorans*, *Bacteroides* sp. *CCUG 39913*, *Swine fecal bacterium* FPC110, Faecalibacterium sp. DJF VR20, *Clostridium methylpentosum*, Oscillospira sp. BA04013493, Candidatus Bacilloplasma, Clostridiales bacterium A2-162, *Coriobacterium* sp. CCUG 33918, *Amphibacillus* sp. YIM-kkny6, *Lachnospiraceae bacterium* DJF RP14, *Clostridium leptum,* and *Ruminococcus* sp. CS1—increase 0.1% T0 vs. T3, *p* = 0.001	Yes / QT (two individuals did not take QT during radiotherapy)
**Wang A et al., 2015** [7] **/** **10.1371/journal.pone.0126312**	Prospective cohort	N = 11 (2:9) Control: N = 4 / 41–65 years (median 51) / Cervical, anal, and colorectal cancer	Pelvic RT / 44–50 Gy 1.8–2.0 Gy/day 5 times a week 5 week period 25 fractions	16s rRNA V3 region / SILVA ribosomal RNA database/MOTHUR	Fecal 2 samples: - Immediately before - Just after RT	- Shannon index alfa diversity—deterioration - Firmicutes-to-Bacteroidetes ratio decreased from 1.79 to 0.83 in patients without diarrhea and from 2.15 to 0.63 in patients with diarrhea - Unclassified bacteria significantly increased in patients who developed diarrhea, but not in those who did not (to 2.47% vs. 0.88%, respectively) - *Bacteroides*—significantly increased *p* < 0.01 - *Clostridium_XIVa*—significantly increased, *p* < 0.01 no diarrhea and *p* < 0.05 diarrhea - Bacteroidetes—increase - Firmicutes—decrease - *Faecalibacterium and Lachnospiracea—*decreased, *p < 0.01* - *Oscillibacter and Streptococcus*—decreased in nondiarrhea group, *p* < 0.01 - *Roseburia* decreased (*p < 0.05*); *Clostridium* XI and XVIII increased in diarrhea p<0.01 and decreased in nondiarrhea *p* < 0.05 - Unclassified (others)—increased with diarrhea *p* < 0.01 and decreased in nondiarrhea - *Veilonella* decreased with diarrhea *p* < 0.01 and increased in nondiarrhea group *p* < 0.01 - *Sutterella* increased in nondiarrhea group *p* < 0.05 and decreased in diarrhea group *p* < 0.05	Yes / Other exclusion criteria: chemotherapy, steroid, immunosuppressor 1 month before / Comparison between patients that developed diarrhea and those who did not
**Yi et al., 2021** [17] **/** **10.1158/1078-0432.CCR-20-3445**	Prospective cohort	N = 84 (58:26) Control N = 31 / Nonresponder group 56.46 ± 9.47 years Responder 56.64 ± 10.43 / Locally advanced rectal cancer	Pelvic RT / 45–50 Gy daily fraction 1.8–2 Gy	16S rRNA gene V3–V4 region / Ribosomal Database Project classifier /Illumina Miseq / VSearch; USearch STAMP	Fecal / 2 samples :- Initial day (n = 84) - Within three days upon completion of (n = 83) nCRT treatment	- Pre-nCRT samples—significantly higher diversity and a trend towards higher unevenness than the post-nCRT samples - Locally advanced rectal cancer-related bacteria/pathogenic bacteria—decreased - Richness index—decreased, *p* = 0.025 - Chao1 index—decreased, *p* = 0.028 - Simpson index—increased, *p* = 0.38 - *Peptostreptococcus,* Inconsistencies found in the article: decreased, *p* < 0.01 (figure)/in the text were reported to be significantly increased following nCRT - *Parvimonas* and *Porphyromonas* were significantly increased following nCRT - *Faecalibacterium* reduced, *p* < 0.01 - *Streptococcus* increased, *p* < 0.01, exclusively found in the response group - *Oscillibacter* and *Bacteroides* decreased, *p* < 0.05 - *Fusobacterium* significantly decreased, *p* < 0.01 - *Lactobacillus* increased, *p* < 0.05	No / Exclusion: exposure to prebiotics, probiotics, steroids, or immunosuppressants / QTconcurrent
**Wang Z et al., 2019** [26] **/** **10.1111/jcmm.14289**	Prospective cohort	N = 18 (0:18) / 30–67 years (median 57) / Cervical cancer	Pelvic RT / 50.4 Gy 180cGy/fraction	16s rRNA / Illumina Hiseq / QIIME / UPARSE / Greengene database	Fecal / 2 samples: - One day before - First day after the treatment	- 595 distinct OTUs were shared by all the RE patients over irradiation, 180 distinguished pre-RT patients and 58 distinguished post-RT patients - Simpson and Shannon indices—decreased, but no significant difference - *Prevotella_9*—decreased - *Bacteroides*—decreased - *Serratia*—increased - *Roseburia*—increased - *Prevotella_2*—increased - *Citrobacter*—decreased - *Megamonas*—increased - *Coprococcus*—decreased significantly, *p* = 0.034	Yes / Other exclusion criteria:recent use of probiotics; proton pump inhibitors; other morbidities such as enteritis or autoimmune condition
**Sahly et al., 2019** [27] **/** **10.7717/peerj.7683**	Prospective cohort	N = 3 (3:0) Control N = 2 / 3.5–7 years / Rhabdomyosarcoma near pelvic region	Pelvic RT / 50.4 Gy 180 cGy / fraction 28 fractions	16s rRNA V3–V5 / Illumina Miseq / QIIME 2 / SILVA database	Fecal / 3 samples: - Before radiotherapy - 12–16 days after - 26–28 days after	- Alpha diversity generally decreased when compared to the mid-point after 12–15 fractions and before radiation but did not indicate a direct relationship. Patients 1 and 2 showed an increase after two exposures, but patient 3 showed a massive decrease. - Firmicutes—decreased - Proteobacteria, Actinobacteria, and Bacteroidetes—increased - *Defluviitaleaceae* and *Ruminococcaceae*—increased - *Clostridiales*, *Bacteroides*, *Streptococcus*, *Dorea*, *Subdoligranulum*, and *Escherichia–Shigella*—increased	No / QT weeks before RT
**Shi et al., 2020** [16] **/** **10.3389/fcimb.2020.562463**	Prospective cohort	N = 22 (16:6) / 45–72 years (median 61) / Rectal cancer	Pelvic RT / 50Gy 2Gy daily fractions	16s rRNA V3–4 region / MOTHUR / SILVA database / Ribosomal Database project	Fecal samples / 2 samples: - At treatment initiation - Just after nCRT	- Richness and diversity were unchanged - *Splanchnicus* increased - *Micrococcaceae* increased after nCRT by both STAMP and LefSe - *Micrococcaceae* increased, *p* = 0.044 - *Rothia* increased, *p* = 0.044 - *Ruminococcus* decreased, *p* = 0.011 - *Fusicatenibacter* decreased, *p* = 0.018 - *Peptostreptococcus* decreased, *p* = 0.034 - *Anaerofilum* decreased, *p* = 0.042 - *Faecalibacterium prausnitzii* decreased, *p* = 0.044 - *Fusicatenibacter saccharivorans* decreased, *p* = 0.016 - *Odoribacter splanchnicus* increased, *p* = 0.036 - *Peptostreptococcus*/unclassified (OTU 00087) decreased, *p* = 0.027 - *Lachnospiraceae*/unclassified (OTU 00192) decreased, *p* = 0.049	Yes / concurrent chemotherapy / Exclusion criteria: steroids and immunosuppressants within the previous 6 months
**Mitra et al., 2020**[18] **/** **10.1016/j.ijrobp.2019.12.040**	Prospective cohort	N = 35 (0:35) / 35–72 years (median 47) / Cervical cancer	Pelvic RT / No information found about doses	16s rRNA V4 region / Illumina MiSeq / SILVA database / UPARSE	Fecal / 4 samples: - Before RT - During radiation therapy (weeks 1, 3, and 5)	- Shannon diversity index α-diversity decreased over the course of radiation—2.9 ± 0.5 at baseline to 2.49 ± 0.7 at week 5, *p* = 0.012 - Barnesiella increased - Clostridiales decreased - Pasteurales increased - Fusobacillales increased - Lactobacillales increased	No / QT Weekly cisplatin
**El Alam et al., 2021**[19] **/** **10.1371/journal.pone.0247905**	Prospective cohort	N = 58 (50:8) / Mean 49.36 ± 10.52 years / Gynecologic cancer patients (55 cervical, 2 vulvar, and 1 with vaginal cancer)	45 Gy (minimum radiation dose) 5 weeks 25 fractions / Either 2 or 5 pulsed dose brachytherapy	16S rRNA V4 region / Alkek Center for Metagenomics and Microbiome Research at Baylor College of Medicine using a methodology from the Human Microbiome Project	Rectal swabs / 5 samples: - Immediately before treatment - 1, 3, 5, and 12 weeks after treatment initiation	- Richness and diversity significantly decreased by week 5 but returned to baseline levels after CRT - Alpha diversity at week 1 or 3, no significant difference, p>0.05. At week 12, samples were not statistically lower than those at baseline (*p* > 0.05 for all). Of the week 5 samples, significant decreases were found compared to baseline samples: - Mean observed OTU decreased—value was 107.58 vs. 83.79, *p* < 0.001 - Mean Shannon diversity index decreased, 2.91 vs. 2.52, *p* < 0.001, but did not differ significantly between week 5 and either week 1 or 3 - Mean Simpson diversity index decreased, 0.87 vs. 0.81, *p* = 0.002, but did not differ significantly between week 5 and either week 1 or 3 - Mean inverse Simpson diversity index decreased, 11.39 vs. 8.23, *p* = 0.001, but did not differ significantly between week 5 and either week 1 or 3 - Mean Fisher diversity index decreased, 18.26 vs. 13.53, *p* < 0.001 - Bacteroidetes decreased. Significant alteration between baseline and week 12 - Proteobacteria increased but tended to return to baseline levels after CRT - *Clostridiales* and *Faecalibacterium* decreased - *Ezakiella* decreased. Between week 5 and week 12 returned to baseline - *Gammaproteobacteria* increased. Between week 5 and week 12 continued to decrease - *Bacilli, Pasteurellales*, *Pasteurellaceae,* and *Haemophilus* increased - *Clostridia* significantly decreased from baseline to week 5 but tended to return to baseline levels after CRT. Most levels of rare Clostridia species were significantly higher at baseline than at week 5. However, a small fraction of individual OTUs of Clostridia increased their occupancy during CRT	No / QTcisplatin and brachytherapy / 53 patients did not provide samples at all time points
**Cuzzolin et al., 1992** [23] **/** **10.1080/1120009x.1992.11739160**	Prospective cohort	N = 15 (0:15) / 45–79 years / Gynecological cancer	Pelvic RT / 4000 cGy in 4 to 5 weeks overall 175–200 c Gy daily 5 days per week	Culture counts / Agar-based methods	Fecal / 5 samples: - 1 before - 4 after irradiation fractions	- *Escherichia coli*—significant decrease after the 3 first RTs, *p* < 0.05 - *Aeromonas hydrophila—*significant decrease after the 2 first RTs, *p* < 0.05 - *Peptococcus* and *Peptostreptococcus* spp. and *Fusobacterium nucleatum*—significant decrease following the first radiation exposure, *p* < 0.05 - *Enterococcus faecium 1; Lactobacilli aerobi* spp.*,* total anaerobes, *Lactobacilli anaerobi spp.*—significant decrease after total dose RT, *p* < 0.05 - *Clostridium* spp. *(Cl. histolyticum, Cl. bifermentans, Cl. sporogenes)—*growth was observed in some patients - Fecal enterotoxin - not influenced by multiple radiations	Yes / Other exclusion criteria: cytotoxic chemotherapy
**Sajjadieh et al., 2012** [24] **/** **PMID: 23400266; PMCID: PMC3564093**	Prospective cohort	N = 75 Control group n = 20 / 4–18 years / Living in a contaminated area near Chernobyl	Ambient radiation / Internal whole-body radioactivity Cs-137 measured by γ-ray spectrometry	Bacterial culture; colony-forming units / CPLX agar Bifidobacterium; LBS agar Lactobacillus; COBA agar Enterococcus; DHL agar Enterobacter	Fecal / 1 sample	- Dysbiosis in 81.3% - *Enterobacter*—inconsistency found in the article between text and tabulated results: significantly decreased compared to control/increased in table, *p* < 0.01 - *Enterococcus*—significantly decreased compared to control, *p* < 0.01 - *Lactobacillus*—significantly decreased compared to control, *p* < 0.01 - *Bifidobacterium*—significantly decreased compared to control, *p* < 0.01	Yes / -
**García-Peris et al., 2012** [25] **/** **10.3305/nh.2012.27.6.5992**	RCT	N = 31 (0:31) / 36–77 years (median 59) / Gynecological cancer	Pelvic RT / 52.2 Gy 1.8 Gy/day 5 times a week 29 sessions	Culture counts / Fluorescent in situ hybridization, genus-specific probes (Bifidobacterium: Bif164 and Lactobacillus: LAC158)	Fecal / 4 samples: - 7 days before RT - 15 days after RT - At the end of the treatment - 3 weeks after RT	- *Lactobacillus*—significant decrease, *p* < 0.05 - *Bifidobacterium*—significant decrease, *p* < 0.05	Yes / Other exclusion criteria: previous RT; previous or adjuvant QT; immunosuppressive
**Nam et al., 2013** [5] **/** **10.1371/journal.pone.0082659**	Prospective cohort	N = 9 (0:9) Control N = 6 / 35–63 years / Gynecologic cancer (cervix and endometrium)	Pelvic RT / 50.4 Gy 5 times a week 5 week period 25 fractions	16S rRNA V1/V2 / QIIME MOTHUR UPARSE / Ribosomal and SILVA databases	Fecal / 4 samples: - 1 week before - After the first RT - At the end of the fifth RT - 1–3 months after final RT	- Shannon index—decrease, *p* = 0.220 - Number of unique sequences—decrease 10.4%, *p* = 0.06 - Estimated OTUs significantly decreased through the radiotherapy, *p* = 0.04 - Actinobacteria and Proteobacteria fluctuating pattern: increased after 1 RT session, decreased after the fifth and in the follow-up samples - Firmicutes steadily decreased 10% through radiotherapy, *p* = 0.09 - Fusobacteria increased 6.0 times, *p* = 0.05 - Bacteroidetes relative abundance gradually decreased during RT, but largely increased at T3 - Unclassified bacteria increased 9.9%, *p* = 0.04 - * Eubacteriaceae* —significantly decreased, *p* < 0.032 - * Fusobacteriaceae* —significantly increased at T2 - * Streptococcaceae* —significantly increased at T1, *p* < 0.05 - * Veillonellaceae* and *Enterococcaceae*, *Lactobacillales bacterium,* and *Butyrate-producing bacterium*—unchanged at T0, T1, and T2. Significant decrease from T0 to T3, *p* = 0.050 - *Ruminococcus*—slightly increased at T1 and eliminated at T2. Three ruminoccocal microorganisms were identified again at T3. In addition, some species-level taxa included in the same genus showed opposite patterns of variation (increment/decrement). - * Ruminococcus* sp. *CO28, Roseburia* sp. *DJF VR77, Ruminococcus* sp. *CO41,* and *Lachnospira pectinoschiza*—increase T0 vs. T1, *p* = 0.001 - * Weissella confuse, Enterobacter* sp. *mcp11b, Klebsiella pneumonia,* and *Adlercreutzia equolifaciens*—decrease 0.1% T0 vs. T1, *p* = 0.001 - * Butyrate-producing bacterium* SS2/1—increase 2.9% T0 vs. T2, *p* = 0.009 - * Ruminococcus callidus* —decrease 1.0% T0 vs. T2, *p* = 0.03 - * Dialister* sp. *E2 20* —decrease 1.0% T0 vs. T2, *p* = 0.013 - * Human intestinal firmicute CB47* —increase 3.5% T0 vs. T2, *p* = 0.025 - * Eubacterium eligens* —decrease 0.7% T0 vs. T2, *p* = 0.032 - * Eubacterium hallii* —decrease 0.1% T0 vs. T2, *p* = 0.041 - * Actinomyces odontolyticus* —decrease 0.1% T0 vs. T2, *p* = 0.046 - * Lactobacillus murinus* —decrease 0.1% T0 vs. T2, *p* = 0.039 - * Clostridiales bacterium DJF CP67* —increase 0.2% T0 vs. T2, *p* = 0.009 - *Clostridium sp. BGC36* was eliminated—decrease 0.1% T0 vs. T3, *p* = 0.001 - * Prevotella stercorea* —decrease 0.3% T0 vs. T3, *p* = 0.001 - *Ruminococcus* sp. DJF VR52, *Prevotella copri*, Ruminococcus sp. CO28, *Butyrate-producing bacterium* T1-815, *Roseburia inulinivorans*, *Bacteroides* sp. *CCUG 39913*, *Swine fecal bacterium* FPC110, Faecalibacterium sp. DJF VR20, *Clostridium methylpentosum*, Oscillospira sp. BA04013493, Candidatus Bacilloplasma, Clostridiales bacterium A2-162, *Coriobacterium* sp. CCUG 33918, *Amphibacillus* sp. YIM-kkny6, *Lachnospiraceae bacterium* DJF RP14, *Clostridium leptum,* and *Ruminococcus* sp. CS1—increase 0.1% T0 vs. T3, *p* = 0.001	Yes / QT (two individuals did not take QT during radiotherapy)
**Wang A et al., 2015** [7] **/** **10.1371/journal.pone.0126312**	Prospective cohort	N = 11 (2:9) Control: N = 4 / 41–65 years (median 51) / Cervical, anal, and colorectal cancer	Pelvic RT / 44–50 Gy 1.8–2.0 Gy/day 5 times a week 5 week period 25 fractions	16s rRNA V3 region / SILVA ribosomal RNA database / MOTHUR	Fecal 2 samples: - Immediately before - Just after RT	- Shannon index alfa diversity—deterioration - Firmicutes-to-Bacteroidetes ratio decreased from 1.79 to 0.83 in patients without diarrhea and from 2.15 to 0.63 in patients with diarrhea - Unclassified bacteria significantly increased in patients who developed diarrhea, but not in those who did not (to 2.47% vs. 0.88%, respectively) - *Bacteroides*—significantly increased *p* < 0.01 - *Clostridium_XIVa*—significantly increased, *p* < 0.01 no diarrhea and *p* < 0.05 diarrhea - Bacteroidetes—increase - Firmicutes—decrease - *Faecalibacterium and Lachnospiracea—*decreased, *p < 0.01* - *Oscillibacter and Streptococcus*—decreased in nondiarrhea group, *p* < 0.01 - *Roseburia* decreased (*p < 0.05*); *Clostridium* XI and XVIII increased in diarrhea p<0.01 and decreased in nondiarrhea *p* < 0.05 - Unclassified (others)—increased with diarrhea *p* < 0.01 and decreased in nondiarrhea - *Veilonella* decreased with diarrhea *p* < 0.01 and increased in nondiarrhea group *p* < 0.01 - *Sutterella* increased in nondiarrhea group *p* < 0.05 and decreased in diarrhea group *p* < 0.05	Yes / Other exclusion criteria: chemotherapy, steroid, immunosuppressor 1 month before / Comparison between patients that developed diarrhea and those who did not
**Yi et al., 2021** [17] **/** **10.1158/1078-0432.CCR-20-3445**	Prospective cohort	N = 84 (58:26) Control N = 31 / Nonresponder group 56.46 ± 9.47 years Responder 56.64 ± 10.43 / Locally advanced rectal cancer	Pelvic RT / 45–50 Gy daily fraction 1.8–2 Gy	16S rRNA gene V3–V4 region / Ribosomal Database Project classifier / Illumina Miseq / VSearch; USearch STAMP	Fecal / 2 samples: - Initial day (n = 84) - Within three days upon completion of (n = 83) nCRT treatment	- Pre-nCRT samples—significantly higher diversity and a trend towards higher unevenness than the post-nCRT samples - Locally advanced rectal cancer-related bacteria/pathogenic bacteria—decreased - Richness index—decreased, *p* = 0.025 - Chao1 index—decreased, *p* = 0.028 - Simpson index—increased, *p* = 0.38 - *Peptostreptococcus,* Inconsistencies found in the article: decreased, *p* < 0.01 (figure)/in the text were reported to be significantly increased following nCRT - *Parvimonas* and *Porphyromonas* were significantly increased following nCRT - *Faecalibacterium* reduced, *p* < 0.01 - *Streptococcus* increased, *p* < 0.01, exclusively found in the response group - *Oscillibacter* and *Bacteroides* decreased, *p* < 0.05 - *Fusobacterium* significantly decreased, *p* < 0.01 - *Lactobacillus* increased, *p* < 0.05	No / Exclusion: exposure to prebiotics, probiotics, steroids, or immunosuppressants /QT concurrent
**Wang Z et al., 2019** [26] **/** **10.1111/jcmm.14289**	Prospective cohort	N = 18 (0:18) / 30–67 years (median 57) / Cervical cancer	Pelvic RT / 50.4 Gy 180cGy/fraction	16s rRNA / Illumina Hiseq / QIIME / UPARSE / Greengene database	Fecal / 2 samples:- One day before - First day after the treatment	- 595 distinct OTUs were shared by all the RE patients over irradiation, 180 distinguished pre-RT patients and 58 distinguished post-RT patients - Simpson and Shannon indices—decreased, but no significant difference - *Prevotella_9*—decreased - *Bacteroides*—decreased - *Serratia*—increased - *Roseburia*—increased - *Prevotella_2*—increased - *Citrobacter*—decreased - *Megamonas*—increased - *Coprococcus*—decreased significantly, *p* = 0.034	Yes / Other exclusion criteria: recent use of probiotics; proton pump inhibitors; other morbidities such as enteritis or autoimmune condition
**Sahly et al., 2019** [27] **/** **10.7717/peerj.7683**	Prospective cohort	N = 3 (3:0) Control N = 2 /3.5–7 years / Rhabdomyosarcoma near pelvic region	Pelvic RT / 50.4 Gy 180 cGy/fraction 28 fractions	16s rRNA V3–V5 / Illumina Miseq / QIIME 2 / SILVA database	Fecal / 3 samples: - Before radiotherapy - 12–16 days after - 26–28 days after	- Alpha diversity generally decreased when compared to the mid-point after 12–15 fractions and before radiation but did not indicate a direct relationship. Patients 1 and 2 showed an increase after two exposures, but patient 3 showed a massive decrease. - Firmicutes—decreased - Proteobacteria, Actinobacteria, and Bacteroidetes—increased - *Defluviitaleaceae* and *Ruminococcaceae*—increased - *Clostridiales*, *Bacteroides*, *Streptococcus*, *Dorea*, *Subdoligranulum*, and *Escherichia–Shigella*—increased	No / QT weeks before RT
**Shi et al., 2020** [16] **/** **10.3389/fcimb.2020.562463**	Prospective cohort	N = 22 (16:6) / 45–72 years (median 61) / Rectal cancer	Pelvic RT / 50Gy 2Gy daily fractions	16s rRNA V3–4 region / MOTHUR / SILVA database / Ribosomal Database project	Fecal samples / 2 samples: - At treatment initiation - Just after nCRT	- Richness and diversity were unchanged - *Splanchnicus* increased - *Micrococcaceae* increased after nCRT by both STAMP and LefSe - *Micrococcaceae* increased, *p* = 0.044 - *Rothia* increased, *p* = 0.044 - *Ruminococcus* decreased, *p* = 0.011 - *Fusicatenibacter* decreased, *p* = 0.018 - *Peptostreptococcus* decreased, *p* = 0.034 - *Anaerofilum* decreased, *p* = 0.042 - *Faecalibacterium prausnitzii* decreased, *p* = 0.044 - *Fusicatenibacter saccharivorans* decreased, *p* = 0.016 - *Odoribacter splanchnicus* increased, *p* = 0.036 - *Peptostreptococcus*/unclassified (OTU 00087) decreased, *p* = 0.027 - *Lachnospiraceae*/unclassified (OTU 00192) decreased, *p* = 0.049	Yes / concurrent chemotherapy / Exclusion criteria: steroids and immunosuppressants within the previous 6 months
**Mitra et al., 2020**[18] **/** **10.1016/j.ijrobp.2019.12.040**	Prospective cohort	N = 35 (0:35) / 35–72 years (median 47) / Cervical cancer	Pelvic RT / No information found about doses	16s rRNA V4 region / Illumina MiSeq / SILVA database / UPARSE	Fecal / 4 samples: - Before RT - During radiation therapy (weeks 1, 3, and 5)	- Shannon diversity index α-diversity decreased over the course of radiation—2.9 ± 0.5 at baseline to 2.49 ± 0.7 at week 5, *p* = 0.012 - Barnesiella increased - Clostridiales decreased - Pasteurales increased - Fusobacillales increased - Lactobacillales increased	No / QT Weekly cisplatin
**El Alam et al., 2021**[19] **/** **10.1371/journal.pone.0247905**	Prospective cohort	N = 58 (50:8) / Mean 49.36 ± 10.52 years / Gynecologic cancer patients (55 cervical, 2 vulvar, and 1 with vaginal cancer)	45 Gy (minimum radiation dose) 5 weeks 25 fractions / Either 2 or 5 pulsed dose brachytherapy	16S rRNA V4 region / Alkek Center for Metagenomics and Microbiome Research at Baylor College of Medicine using a methodology from the Human Microbiome Project	Rectal swabs / 5 samples: - Immediately before treatment - 1, 3, 5, and 12 weeks after treatment initiation	- Richness and diversity significantly decreased by week 5 but returned to baseline levels after CRT - Alpha diversity at week 1 or 3, no significant difference, p>0.05. At week 12, samples were not statistically lower than those at baseline (*p* > 0.05 for all). Of the week 5 samples, significant decreases were found compared to baseline samples: - Mean observed OTU decreased—value was 107.58 vs. 83.79, *p* < 0.001 - Mean Shannon diversity index decreased, 2.91 vs. 2.52, *p* < 0.001, but did not differ significantly between week 5 and either week 1 or 3 - Mean Simpson diversity index decreased, 0.87 vs. 0.81, *p* = 0.002, but did not differ significantly between week 5 and either week 1 or 3 - Mean inverse Simpson diversity index decreased, 11.39 vs. 8.23, *p* = 0.001, but did not differ significantly between week 5 and either week 1 or 3 - Mean Fisher diversity index decreased, 18.26 vs. 13.53, *p* < 0.001 - Bacteroidetes decreased. Significant alteration between baseline and week 12 - Proteobacteria increased but tended to return to baseline levels after CRT - *Clostridiales* and *Faecalibacterium* decreased - *Ezakiella* decreased. Between week 5 and week 12 returned to baseline - *Gammaproteobacteria* increased. Between week 5 and week 12 continued to decrease - *Bacilli, Pasteurellales*, *Pasteurellaceae,* and *Haemophilus* increased - *Clostridia* significantly decreased from baseline to week 5 but tended to return to baseline levels after CRT. Most levels of rare Clostridia species were significantly higher at baseline than at week 5. However, a small fraction of individual OTUs of Clostridia increased their occupancy during CRT	No / QT cisplatin and brachytherapy / 53 patients did not provide samples at all time points

**Table 5:** Risk of bias—prospective cohorts.

Author, Year	Selection	Comparability	Outcome	Score
Cuzzolin et al., 1992 [23]	*0**	*0	*0*	5/9
Sajjadieh et al., 2012 [24]	*00*	*0	*0*	5/9
Nam et al., 2013 [5]	****	*0	***	8/9
Wang A et al., 2015 [7]	*0**	*0	*0*	6/9
Yi et al., 2021 [17]	****	*0	***	8/9
Wang Z et al., 2019 [26]	****	*0	*0*	7/9
Sahly et al., 2019 [27]	****	*0	*0*	7/9
Shi et al., 2020 [16]	****	**	*0*	8/9
Mitra et al., 2020 [18]	****	**	*0*	8/9
El Alam et al., 2021 [19]	****	*0	**0	7/9

Note: *, yes; 0, no.

**Table 6:** Risk of bias of the randomized controlled trial—García-Peres et al., 2012 [25].

Domain	Risk of Bias	Comments
Sequence generation	High	No information regarding the sequence generation. “patients were randomised to receive...”
Allocation concealment	Low	“coded sachets”
Blinding of participants, personnel, and outcome assessors	Low	Outcome assessors and participants blinded
Incomplete outcome data	Low	“Nine patients were excluded from the study: four because they were prescribed antibiotics, three for personal reasons, and two due to lack of adherence”
Selection outcome reporting	Low	Study protocol available and all of study’s pre-specified outcomes have been reported
Other sources of bias	Low	Study appears to be free of other sources of bias

**Table 7:** Key findings from selected studies.

Dysbiosis	
- Was found in 81.3% (Sajjadieh et al., 2012) [24]	
**Diversity**	
**Alpha diversity**	
Alpha diversity	- Generally **decreased** but did not indicate a direct relationship. Patients 1 and 2 exhibited an **increase** after two exposures, whereas patient 3 exhibited a massive drop (Sahly et al., 2019) [27] - Significantly **decreased** by week 5 but returned to baseline levels after CRT. Week 1 or 3—no significant difference (*p* > 0.05). At week 12, levels were not statistically lower than those at baseline (*P* > 0.05) (El Alam et al., 2021) [19]
Shannon index	- **Deterioration** (Wang A et al., 2015) [7] - **Decreased** (2.9 ± 0.5 at baseline to 2.49 ± 0.7 at week 5; *p* = 0.012 (Mitra A et al., 2019) [18] - **Decreased**, no significant difference (Wang Z et al., 2019) [26] - **Decrease**, *p* = 0.220 (Nam et al., 2013) [5] - **Decreased** from baseline to week 5 2.91 vs. 2.52, *p* < 0.001; did not differ significantly between week 5 and either week 1 or 3 (El Alam et al., 2021) [19]
Simpson index	- **Increased**, *p* = 0.38 (Yi Y, 2021) [17] - **Decreased, no significant difference** (Wang Z et al., 2019) [26] - **Decreased** from baseline to week 5: 0.87 vs. 0.81, *p* = 0.002, but did not differ significantly between week 5 and either week 1 or 3 (El Alam et al., 2021) [19]
**Beta diversity**	
- **Shifts** from baseline through the end of radiation treatment were also observed, *p* = 0.04 (Mitra et al., 2019) [18]	
**Richness**	
Richness index	- **Decreased**, *p* = 0.025 (Yi et al., 2021) [17]
Chao1 index	- **Decreased**, *p* = 0.028 (Yi et al., 2021) [17]
OTUs	- **Reduced** unique sequences (*p* = 0.06) and estimated OTUs decreased, *p* = 0.364 (Nam et al., 2013) [5] - 595 distinct **OTUs** were shared by all the RE patients over irradiation, 180 distinguished pre-RT patients and 58 distinguished post-RT patients (Wang Z et al., 2019) [26] - **Decreased** baseline vs. week 5: 107.58 vs. 83.79 (*p* < 0.001) (El Alam et al., 2021) [19]
**Composition**	
**Phylum level**	
Firmicutes/Bacteroidetes ratio	- **Decreased** from 1.79 to 0.83 in patients without diarrhea and from 2.15 to 0.63 in patients with diarrhea (Wang A et al., 2015) [7]
Unclassified bacteria	- Significant **increase** in patients who developed diarrhea, but not in those who did not (Wang A et al., 2015) [7] - **Increased** 9.9%, *p* = 0.04 (Nam et al., 2013) [5]
Actinobacteria	- **Fluctuating** pattern—increase after first radiotherapy session and **decrease** after fifth and in follow-up (Nam et al., 2013) [5] - **Increased** (Sahly et al., 2019) [27]
Bacteroidetes	- **Decreased** during radiation therapy but was largely increased at T3 (Nam et al., 2013) [5] - **Increased** (Sahly et al., 2019) [27] - **Increased** (Wang A et al., 2015) [7] - **Decreased** (El Alam et al., 2021) [19]
Firmicutes	- **Decreased** 10.1% through radiation (Nam et al., 2013) [5] - **Decreased** (Sahly et al., 2019) [27] - **Decreased** (Wang A et al., 2015) [7]
Fusobacteria	- **Increased** 6.0 times higher at T2 (*p* = 0.05). (Nam et al., 2013) [5]
Proteobacteria	- **Fluctuating** pattern—**increase** after 1 and **decreased** after the fifth and follow-up (Nam et al., 2013) [5] - **Increased** (Sahly et al., 2019) [27] - **Increased**. Tended to return to baseline levels after CRT (El Alam et al., 2021) [19]
**Class level**	
Gammaproteobacteria	- **Increased**, but returned to normal (El Alam et al., 2021) [19]
Bacilli	- **Increased** (El Alam et al., 2021) [19]
Clostridia	- Significantly **decreased** from baseline to week 5 but tended to return to baseline levels after CRT. However, a small fraction of individual OTUs of Clostridia increased during CRT (El Alam et al., 2021) [19]
**Order level**	
Clostridiales	- **Decreased** (Mitra et al., 2020) [18] - **Decreased** (El Alam et al., 2021) [19] - **Increased** (Sahly et al., 2019) [27]
Lactobacillales	- **Increased** (Mitra et al., 2020) [18]
Fusobacteriales	- **Increased** (Mitra et al., 2020) [18]
Pasteurellales	- **Increased** (Mitra et al., 2020) [18] - **Increased** (El Alam et al., 2021) [19]
**Family level**	
* Defluviitaleaceae *	- **Increased** (Sahly et al., 2019) [27]
* Eubacteriaceae *	- **Decreased**, *p* < 0.032 (Nam et al., 2013) [5]
* Fusobacteriaceae *	- Significantly **increased** at T2 (Nam et al., 2013) [5]
*Lachnospiracea*	- **Decreased**, *p* < 0.01 (Wang A et al., 2015) [7]
* Streptococcaceae *	- Significantly **increased** at T1 (Nam et al., 2013) [5]
* Veillonellaceae *	- **Decreased** T0 and T3, *p* = 0.05. **Unchanged** at T0, T1, and T2 (Nam et al., 2013) [5]
* Enterococcaceae *	- **Decreased** T0 and T3, *p* = 0.05. **Unchanged** at T0, T1, and T2 (Nam et al., 2013) [5]
* Pasteurellaceae *	- **Increased** (El Alam et al., 2021) [19]
* Ruminococcaceae *	- **Increased** (Sahly et al., 2019) [27]
**Genus level**	
*Bacteroides*	- Significantly **increased**, *p* < 0.01 (Wang A et al., 2015) - **Increased** (Sahly et al., 2019) [27] - **Decreased**, *p* < 0.05 (Yi et al., 2021) [17] - **Decreased** (Wang Z et al., 2019) [26]
*Bifidobacterium*	- Significant **decrease**, *p* < 0.05 (García-Peris et al., 2012) [25] - Significant **decrease**, *p* < 0.01 (Sajjadieh et al., 2012) [24]
*Citrobacter*	- **Decreased** (Wang Z et al., 2019) [26]
*Clostridium_XIVa*	- Significant **increase**, *p* < 0.01 no diarrhea and *p* < 0.05 with diarrhea (Wang A et al., 2015) [7]
*Clostridium* XI and XVIII and unclassified (others)	- **Increased**, *p* < 0.01, in patients with diarrhea and **decreased**, *p* < 0.05, in nondiarrhea group (Wang A et al., 2015) [7]
*Coprococcus*	- Significant **decrease**, *p* = 0.034 (Wang Z et al., 2019) [26]
*Dorea*	- **Increased** (Sahly et al., 2019) [27]
*Enterobacter*	- Inconsistencies in the article. Significant **decrease** *p* < 0.01/**increase** (Sajjadieh et al., 2012)
*Enterococcus*	- Significant **decrease**, *p* < 0.01 (Sajjadieh et al., 2012) [24]
*Escherichia–Shigella*	- **Increased** (Sahly et al., 2019) [27]
*Ezakiella*	- **Decreased**. Between week 5 and week 12 returned to baseline (El Alam et al., 2021) [19]
*Fusobacterium*	- Significant **decrease**, p < 0.01 (Yi Y, 2021) [17]
*Faecalibacterium*	- **Decreased**, *p* < 0.01 (Wang A et al., 2015) [7] - **Decreased**, *p* < 0.01 (Yi et al., 2021) [17] - **Decreased** (El Alam MB et al., 2021) [19]
*Haemophilus*	- **Increased** (El Alam MB et al., 2021) [19]
*Lactobacillus*	- Significant **decrease**, *p* < 0.05 (García-Peris et al., 2012) [25] - Significant **decrease**, *p* < 0.01 (Sajjadieh et al., 2012) [24] - **Increased**, *p* < 0.05 (Yi et al., 2021) [17]
*Megamonas*	- **Increased** (Wang Z et al., 2019) [26]
*Oscillibacter*	- **Decreased**, *p* < 0.01 no diarrhea (Wang A et al., 2015) [7] - **Decreased**, *p* < 0.05 (Yi et al., 2021) [17]
*Parvimonas*	- Significant **increase** (Yi et al., 2021) [17]
*Peptostreptococcus*	- Inconsistencies found in the article: significant **increase/decrease** (Yi et al., 2021) [17]
*Porphyromonas*	- Significant **increase** (Yi et al., 2021) [17]
*Roseburia*	- **Decreased**, *p* < 0.05 (Wang A et al., 2015) [7] - **Increased** (Wang Z et al., 2019) [26]
*Ruminococcus*	- Slightly **increased** at T1 and **eliminated** at T2, but three ruminoccocal microorganisms were identified again at T3. In addition, some species-level taxa included in the same genus showed opposite patterns of variation (**increment/decrement**) (Nam et al., 2013) [5]
*Serratia*	- **Increased** (Wang Z et al., 2019) [26]
*Streptococcus*	- **Decreased** (Wang A et al., 2015) [7] - **Increased***p* < 0.01, exclusively found in the R group (Yi et al., 2021) [17] - **Increased** (Sahly et al., 2019) [27]
*Subdoligranulum*	- **Increased** (Sahly et al., 2019) [27]
*Sutterella*	- **Increased**, *p* < 0.05, in nondiarrhea group and **decreased**, *p* < 0.05, in patients with diarrhea (Wang A et al., 2015) [7]
*Veilonella*	- **Decreased**, *p* < 0.01, in patients with diarrhea and **increased**, *p* < 0.01, in nondiarrhea group (Wang A et al., 2015) [7]
*Prevotella_2*	- **Increased** (Wang Z et al., 2019) [26]
*Prevotella_9*	- **Decreased** (Wang Z et al., 2019) [26]
**Species level**	
*Actinomyces odontolyticus*	- **Decrease** 0.1% T0 vs. T2, *p* = 0.046 (Nam et al., 2013) [5]
*Adlercreutzia equolifaciens*	- **Decrease** 0.1% T0 vs. T1, *p* = 0.001 (Nam et al., 2013) [5]
*Aeromonas hydrophila*	- Significant **decrease** after the 2 first RTs, *p* < 0.05 (Cuzzolin, 1992) [23]
*Amphibacillus* sp. *YIM-kkny6*	- **Increase** 0.1% T0 vs. T3, *p* = 0.001 (Nam et al., 2013) [5]
*Bacteroides* sp. *CCUG 39913*	- **Increase** 0.2% T0 vs. T3, *p* = 0.001 (Nam et al., 2013) [5]
*Butyrate-producing* *bacterium T1–815*	- **Increase** 0.2% T0 vs. T3, *p* = 0.001 (Nam et al., 2013) [5]
* Butyrate-producing bacterium *	- At T0, T1, and T2—**unchanged**; at T0 and T3—significant **decrease**, *p* = 0.05 (Nam et al., 2013) [5]
*Butyrate-producing* *bacterium SS2/1*	- **Increase** 2.9% T0 vs. T2, *p* = 0.009 (Nam et al., 2013) [5]
*Candidatus Bacilloplasma*	- **Increase** 0.1% T0 vs. T3, *p* = 0.001 (Nam et al., 2013) [5]
*Coriobacterium* sp. *CCUG 33918*	- **Increase** 0.1% T0 vs. T3, *p* = 0.001 (Nam et al., 2013) [5]
*Clostridium methylpentosum*	- **Increase** 0.1% T0 vs. T3, *p* = 0.001 (Nam et al., 2013) [5]
*Clostridiales bacterium DJF CP67*	- **Increase** 0.2% T0 vs. T2, *p* = 0.009 (Nam et al., 2013) [5]
*Clostridium leptum*	- **Increase** 0.1% T0 vs. T3, *p* = 0.001 (Nam et al., 2013) [5]
*Clostridiales bacterium A2–162*	- **Increase** 0.1% T0 vs. T3, *p* = 0.001 (Nam et al., 2013) [5]
*Clostridium* sp. *BGC36*	- **Eliminated** 0.1% T0 vs. T3, *p* = 0.001 (Nam et al., 2013) [5]
*Clostridium* spp. *(Cl. histolyticum, Cl. bifermentans, Cl. sporogenes)*	- **Growth** was observed in some patients (Cuzzolin, 1992) [23]
*Dialister* sp. *E2 20*	- **Decrease** 1.0% T0 vs. T2 *p* = 0.013 (Nam et al., 2013) [5]
*Escherichia coli*	- Significant **decrease** after the 3 first RTs, *p* < 0.05 (Cuzzolin, 1992) [23]
*Eubacterium eligens*	- **Decrease** 0.7% T0 vs. T2, *p* = 0.032 (Nam et al., 2013) [5]
*Eubacterium hallii*	- **Decrease** 0.1% T0 vs. T2, *p* = 0.041(Nam et al., 2013) [5]
*Enterococcus faecium 1*	- Significant **decrease** after total dose RT, *p* < 0.05 (Cuzzolin, 1992)
*Enterobacter* sp. *mcp11b*	- **Decrease** 0.2% T0 vs. T1, *p* = 0.001 (Nam et al., 2013) [5]
*Fusobacterium nucleatum*	- Significant **decrease** after the first RT, *p* < 0.05 (Cuzzolin, 1992) [23]
*Faecalibacterium Prausnitzii*	- **Decreased**, *p* = 0.044 (Shi et al., 2020) [16]
*Faecalibacterium* sp. *DJF VR20*	- **Increase** 0.2% T0 vs. T3, *p* = 0.001 (Nam et al., 2013) [5]
*Human intestinal firmicute CB47*	- **Increase** 3.5% T0 vs. T2, *p* = 0.025 (Nam et al., 2013) [5]
*Klebsiella pneumonia*	- **Decrease** 0.1% T0 vs. T1, *p* = 0.001 (Nam et al., 2013) [5]
*Lactobacillus murinus*	- **Decrease** 0.1% T0 vs. T2, *p* = 0.039 (Nam et al., 2013) [5]
*Lachnospiraceae bacterium DJF RP14*	- **Increase** 0.1% T0 vs. T3, *p* = 0.001 (Nam et al., 2013) [5]
*Lachnospira pectinoschiza*	- **Increase** 0.1% T0 vs. T1, *p* = 0.001 (Nam et al., 2013) [5]
* Lactobacillales bacterium *	- At T0, T1, and T2—**unchanged**; at T0 and T3—significant differences, *p* = 0.05 (Nam et al., 2013) [5]
*Lactobacilli aerobi* spp.	- Significant **decrease** after total dose RT, *p* < 0.05 (Cuzzolin, 1992) [23]
*Lactobacilli anaerobi* spp.	- Significant **decrease** after total dose RT, *p* < 0.05 (Cuzzolin, 1992) [23]
*Oscillospira* sp. *BA04013493*	- **Increase** 0.1% T0 vs. T3, *p* = 0.001 (Nam et al., 2013) [5]
*Prevotella stercorea*	- **Decrease** 0.3% T0 vs. T3, *p* = 0.001 (Nam et al., 2013) [5]
*Prevotella copri*	- **Increase** 0.3% T0 vs. T3, *p* = 0.001 (Nam et al., 2013) [5]
*Peptococcus and Peptostreptococcus* spp.	- Significant **decrease** following the first radiation exposure, *p* < 0.05 (Cuzzolin, 1992) [23]
*Roseburia inulinivorans*	- **Increase** 0.2% T0 vs. T3, *p* = 0.001 (Nam et al., 2013) [5]
*Ruminococcus* sp. *DJF VR52*	- **Increase** 0.6% T0 vs. T3, *p* = 0.001 (Nam et al., 2013) [5]
*Ruminococcus* sp. *CO28*	- **Increase** 0.4% T0 vs. T1, *p* = 0.001 (Nam et al., 2013) [5]
*Roseburia* sp. *DJFVR77*	- **Increase** 0.3% T0 vs. T1, *p* = 0.001 (Nam et al., 2013) [5]
*Ruminococcus* sp. *CO41*	- **Increase** 0.2% T0 vs. T1, *p* = 0.001 (Nam et al., 2013) [5]
*Ruminococcus callidus*	- **Decrease** 1.0% T0 vs. T2, *p* = 0.03 (Nam et al., 2013) [5]
*Ruminococcus* sp. *CO28*	- **Increase** 0.3% T0 vs. T3, *p* = 0.001 (Nam et al., 2013) [5]
*Ruminococcus* sp. *CS1*	- **Increase** 0.1% T0 vs. T3, *p* = 0.001 (Nam et al., 2013) [5]
*Swine fecal bacterium FPC110*	- **Increase** 0.2% T0 vs. T3, *p* = 0.001 (Nam et al., 2013) [5]
*Weissella confuse*	- **Decrease** 0.3% T0 vs. T1, *p* = 0.001 (Nam et al., 2013) [5]

## Supplementary Materials

The following are available online at www.mdpi.com/article/10.3390/nu13093025/s1, Table S1: PRISMA Checklist for Preferred Reporting Items for Systematic Reviews and Meta-Analyses.
